# Nomogram for predicting pathological complete response to neoadjuvant chemoimmunotherapy in patients with resectable non-small cell lung cancer

**DOI:** 10.3389/fcell.2025.1679782

**Published:** 2025-09-22

**Authors:** Wenyi Liu, Zhilin Sui, Chunguang Wang, Youjun Deng, Songhua Cai, Ran Jia, Zhentao Yu, Mingqiang Kang, Baihua Zhang

**Affiliations:** ^1^ Department of Thoracic Surgery, Fujian Medical University Union Hospital, Fuzhou, China; ^2^ Department of Thoracic Surgery, National Cancer Center/National Clinical Research Center for Cancer/Cancer Hospital & Shenzhen Hospital, Chinese Academy of Medical Sciences and Peking Union Medical College, Shenzhen, China; ^3^ Department of Thoracic Surgery, Hunan Cancer Hospital, Changsha, China

**Keywords:** non-small cell lung cancer, neoadjuvant chemoimmunotherapy, pathological complete response, nomogram, thoracic surgery, prediction model

## Abstract

**Objectives:**

Neoadjuvant chemoimmunotherapy is increasingly employed in resectable non-small cell lung cancer (NSCLC), with variable pathological complete response (pCR) rates. Currently, no reliable preoperative tool is available for predicting pCR. This study develops a nomogram based on clinical variables to predict pCR and guide individualized surgical decisions.

**Methods:**

We retrospectively analyzed data from 179 NSCLC patients (stages IIB-IIIB) who received neoadjuvant chemoimmunotherapy followed by resection (2019–2022). Variables included demographics, smoking history, comorbidities, treatment details, and pathology. Univariate and multivariate logistic regression identified pCR predictors, which were incorporated to build a nomogram. Performance was assessed via area under the curve (AUC), calibration, and decision curve analysis (DCA).

**Results:**

Of 179 patients, 92 (51.4%) achieved pCR. Multivariate analysis identified independent predictors: non-squamous histology (OR 0.344 (non-squamous vs. squamous), 95% CI 0.151–0.707, p = 0.006), positive family history (OR 10.76 (positive vs. negative), 95% CI 1.903–203.3, p = 0.027), shorter smoking cessation duration (defined as time in days from last cigarette to treatment start) (OR 0.999 (per day), 95% CI 0.999–0.999, p = 0.033), older age (OR 1.053 (per year), 95% CI 1.005–1.106, p = 0.032), and more treatment cycles (OR 1.621 (per cycle), 95% CI 1.007–2.661, p = 0.049). The nomogram showed modest discrimination (AUC 0.709, 95% CI 0.633–0.785), good calibration, and net benefit on DCA, though it has not been externally validated and is limited by single-center data, small sample size, high pCR rate, and skewed demographics (95.5% male, 92.7% smokers), potentially limiting generalizability to diverse populations such as females or non-smokers.

**Conclusion:**

This nomogram, derived from routine clinical data, predicts pCR after neoadjuvant chemoimmunotherapy in NSCLC, offering a tool for thoracic surgeons to optimize treatment and surgical planning, despite its modest discriminative power, by serving as a complementary aid in resource-limited settings where biomarkers may not be readily available. External validation in larger, multi-center cohorts is essential.

## 1 Introduction

Significant progress has been made in the neoadjuvant treatment of lung cancer in recent years, particularly with the advent of immunotherapy, which offers new hope to patients (Forde et al., 2018). Immunotherapy enhances the immune system’s ability to combat tumor cells and has become a cornerstone of lung cancer treatment.

The management of NSCLC has been significantly transformed by the introduction of immune checkpoint inhibitors (ICIs), which have shown remarkable efficacy in various clinical trials. Notably, phase III trials such as CheckMate 816 and KEYNOTE-671 have been pivotal in establishing the role of ICIs in the treatment of NSCLC. These trials have demonstrated that ICIs, when used in combination with chemotherapy, can significantly improve outcomes for patients with resectable NSCLC by increasing the pCR rates and decreasing relapse rates (Spicer et al., 2024; Mayenga et al., 2024; Dunne et al., 2024; Horita et al., 2024). Research indicates that this combination therapy significantly improves overall survival (OS), event-free survival (EFS, defined as the time from randomization to progression, recurrence, or death), pCR rates, and major pathological response (MPR, defined as ≤10% viable tumor cells) rates. Despite its advantages, not all NSCLC patients benefit from neoadjuvant chemoimmunotherapy, underscoring the importance of accurately identifying those likely to respond before initiating treatment.

While biomarkers like programmed death-ligand 1 (PD-L1) expression and tumor mutational burden (TMB) are promising for patient selection (Mok et al., 2019; Doroshow et al., 2019; Doroshow et al., 2021), their routine availability is limited, particularly in resource-constrained settings. Our study focuses on accessible clinical variables to complement these approaches.

Despite ongoing efforts to identify predictive factors and models for the success of neoadjuvant chemoimmunotherapy, a reliable and user-friendly tool for predicting pCR is still lacking in clinical practice (Reck et al., 2022). Accurate pre-treatment identification of patients likely to achieve pCR is vital for improving OS, reducing treatment costs, and avoiding overtreatment (Brahmer et al., 2012).

Nomograms provide a practical and visual means of simplifying multifactorial regression models, enabling clinicians to make individualized prognostic predictions based on a patient’s multidimensional characteristics (Balachandran et al., 2015). Compared to traditional staging or single-factor indicators, nomograms often deliver superior predictive performance (Iasonos et al., 2008). The justification for developing a new nomogram is supported by prior attempts, such as models incorporating radiomics or biomarkers for pCR prediction in neoadjuvant settings, though many lack external validation or rely on inaccessible data (Qu et al., 2024; Han et al., 2024; Hu et al., 2024). Given the high incidence of pCR following neoadjuvant treatment in lung cancer and the limited availability of nomograms for pCR prediction, developing a model that integrates pre- and post-treatment parameters holds significant value in identifying candidates likely to achieve pCR (Shariat et al., 2008).

Based on the above background, this study aims to collect and analyze extensive clinical data from NSCLC patients treated with neoadjuvant chemoimmunotherapy at our hospital. By evaluating multiple variables before and after neoadjuvant treatment, we identify potential predictors of pCR. Using logistic regression analysis, we select relevant parameters to construct a nomogram for pCR prediction, which is subsequently validated internally to assess its performance. Although our model relies on clinical variables rather than molecular biomarkers (due to data availability), it serves as a complementary tool to emerging biomarker-based approaches. While biomarkers like PD-L1 and TMB are promising, their availability is limited in routine practice. This study develops a nomogram using accessible clinical-surgical variables to predict pCR, complementing existing tools. Compared to biomarker-inclusive models (e.g., AUC >0.80 in refs 13–15), our nomogram offers lower-cost accessibility but inferior discrimination, highlighting the need for integration. The sample size (n = 179) is comparable to similar retrospective studies but limits generalizability, particularly without external validation.

## 2 Materials and methods

### 2.1 Patients

A retrospective analysis was conducted using data from NSCLC patients who underwent neoadjuvant treatment followed by lung cancer surgery at Cancer Hospital Chinese Academy of Medical Sciences, Shenzhen Center between 2019 and 2022. Patients were included in the study if they met all of the following criteria: Pathologically diagnosed with NSCLC (including squamous cell carcinoma, adenocarcinoma, or other subtypes) and having complete clinical and pathological data, in accordance with the 8th edition of the AJCC staging system; Pathological staging of IIB-IIIB stage NSCLC patients; Received platinum-based chemotherapy combined with immune checkpoint inhibitors; Postoperative pathological assessment clearly classified as either pCR or non-pCR.

Exclusion criteria included: those who only received neoadjuvant chemotherapy, only received neoadjuvant immunotherapy, interrupted treatment regimens, and those with incomplete data.

**FIGURE 1:**
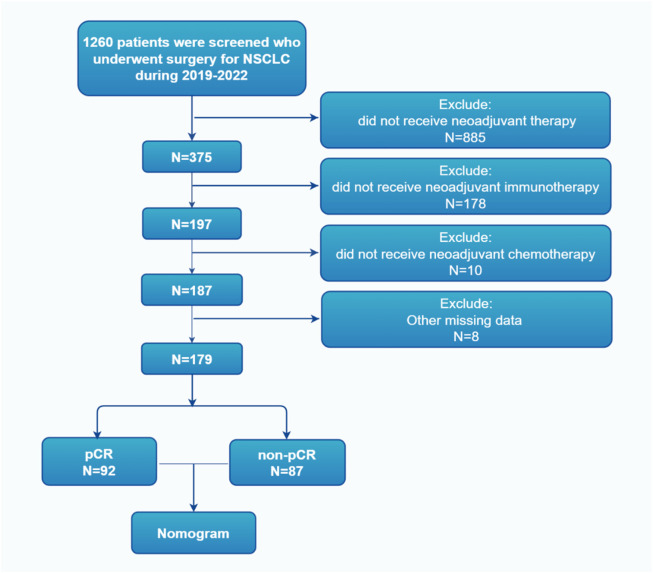
Flowchart of the patient screening process, illustrating inclusion/exclusion criteria and final cohort derivation from 375 initial patients to 179 analyzed cases.

The patient selection process for this study is summarized in Figure 1.

### 2.2 Treatment and follow-up

The neoadjuvant treatment regimens included neoadjuvant chemotherapy combined with immunotherapy. Chemotherapy consisted of albumin-bound paclitaxel or pemetrexed combined with platinum-based drugs, while immunotherapy included pembrolizumab, nivolumab, and other domestically produced immunotherapeutic agents in China. The multidisciplinary team (MDT) determined the specific neoadjuvant treatment regimen and the number of treatment cycles based on each patient’s condition and clinical trial participation.

Following neoadjuvant treatment, all patients underwent radical surgery, which included lobectomy, sleeve lobectomy, or pneumonectomy. Postoperative tumor specimens were evaluated by experienced pathologists. Pathological complete response (pCR) was defined as the complete absence of residual cancer cells in all pathological sections of the resected lung tissue and lymph nodes (ypT0N0). Patients with any residual cancer cells identified in the final pathology were classified as non-pCR.

After surgery, patients underwent routine follow-up: every 3 months during the first 2 years, every 6 months during the subsequent 3 years, and annually thereafter.

### 2.3 Data collection

The selection of variables was based on previously published literature and supplemented by clinical expertise. The following parameters were evaluated as potential predictive factors for pCR: gender, age, presence of symptoms at initial diagnosis, weight loss at initial diagnosis, smoking history, smoking index, smoking cessation duration (time from last cigarette to treatment start, in days), alcohol consumption history, comorbidities, family history of cancer, tumor location, tumor type (central or peripheral), clinical T stage (cT), clinical N stage (cN), clinical TNM stage (cTNM), number of neoadjuvant treatment cycles, type of immunotherapy drug, type of chemotherapy drug, type of platinum-based drug, treatment-related adverse reactions, the interval between the end of neoadjuvant therapy and surgery, and pathological tumor subtype.

Age was stratified into <58 years and ≥58 years based on the X-tile method (optimal cut-off determined by minimizing the chi-square p-value for association with pCR, ensuring balanced subgroups and statistical significance). Comorbidities were categorized as none, hypertension, diabetes, or other conditions. Immunotherapy drugs included pembrolizumab, nivolumab, and other domestically produced immunotherapy agents. Chemotherapy drugs were categorized as albumin-bound paclitaxel or other agents, and platinum-based drugs included cisplatin, carboplatin, or other platinum compounds. Adverse events were classified into none, myelosuppression, hepatic and renal dysfunction, or immune-related adverse reactions, and were graded from 1 to 4 per CTCAE v5. Missing data were minimal (<5% across variables) and handled via complete-case analysis; sensitivity analyses excluding cases with missing values confirmed no significant bias in results.

### 2.4 Statistical analysis

Categorical variables were compared using the chi-square test or Fisher’s exact test for small expected frequencies (<5). Continuous variables were assessed using the independent t-test for normally distributed data or the Mann-Whitney U test for non-normally distributed data. Analyses were performed using SPSS v27 and R v4.1.3. A two-sided p-value of <0.05 was considered statistically significant, with p < 0.1 used as a threshold for including variables in multivariate analysis to avoid omitting potentially relevant predictors while controlling for type I error.

Univariate analysis was performed to retain variables significantly associated with pCR. Significant features (p < 0.1) entered a multivariate logistic regression model to derive the predictive equation for pCR. The final regression coefficients were used to construct a nomogram to quantify the contribution of each variable to pCR prediction. Hosmer-Lemeshow test assessed goodness-of-fit. Nomogram was constructed using the rms package in R. Multicollinearity was checked using variance inflation factor (VIF) (all VIF <5 ranged from 1.2 to 3.8, indicating no significant multicollinearity). Potential confounders and interactions (e.g., age-smoking cessation) were tested; none were significant (p > 0.05 for interaction terms). To mitigate potential overfitting in this small cohort, we employed bootstrap resampling (n = 500) for internal validation; external validation was not feasible due to the single-center retrospective design.

Model evaluation: The area under the receiver operating characteristic (ROC) curve (AUC, measuring discriminative ability) was used to assess the model’s discriminative ability. Calibration curves were used to evaluate the consistency between predicted probabilities and actual outcomes. Bootstrap resampling (n = 500) to validate the robustness and reproducibility of the model, including bias-corrected AUC to address potential overfitting.

## 3 Results

### 3.1 Patient characteristics

From 2019 to 2022, a total of 375 NSCLC patients received neoadjuvant treatment followed by surgery at our institution. After excluding patients who did not meet the inclusion criteria or had incomplete data, 179 patients were included in the final analysis. Among them, 92 patients (51.4%) achieved pCR, while 87 patients (48.6%) were classified as non-pCR.

The majority of the patients were male (171 cases, 95.5%), and over half were aged 58 years or older (100 cases, 55.9%). This male-dominant cohort may bias results toward smoking-related factors common in male populations. Most patients presented with symptoms at diagnosis, such as coughing or shortness of breath (132 cases, 73.7%), and did not experience weight loss (148 cases, 82.7%). Comorbidities were absent in 113 patients (63.1%). Central-type tumors were predominant (143 cases, 79.9%), and most patients had advanced nodal involvement (cN1-2, 168 cases, 93.9%). The tumor stage distribution was as follows: 38 cases (21.2%) were stage IIB, 83 cases (46.4%) were stage IIIA, and 58 cases (32.4%) were stage IIIB.

Neoadjuvant treatment cycles varied, with 107 patients (59.8%) completing 2 cycles, 54 patients (30.2%) completing 3 cycles, and 18 patients (10.1%) completing 4 cycles. Treatment-related adverse events included myelosuppression (49 cases, 27.4%), hepatic and renal dysfunction (44 cases, 24.6%), and immune-related adverse reactions (24 cases, 13.4%) (Table 1).

**TABLE 1:** Patient characteristics and univariate analysis for categorical variables associated with pCR.

Patient characteristics	pCR N = 92	Non-pCR N = 87	p-value	Univariate analysis	
				OR (95% CI)	p-value
Sex, n (%)			0.487	0.553 (0.111–2.324)	0.427
Male	89 (96.74%)	82 (94.25%)			
Female	3 (3.26%)	5 (5.75%)			
Age, Mean ± SD	60.42 ± 6.23	58.89 ± 7.76	0.147	1.032 (0.99–1.078)	0.145
Age stratification, n (%)			0.526	0.79 (0.436–1.425)	0.433
≥58	54 (58.70%)	46 (52.87%)			
<58	38 (41.30%)	41 (47.13%)			
Symptom, n (%)			0.367	1.441 (0.74–2.835)	0.285
No	21 (22.83%)	26 (29.89%)			
Yes	71 (77.17%)	61 (70.11%)			
Weight loss, n (%)			0.823	1.182 (0.544–2.604)	0.673
No	75 (81.52%)	73 (83.91%)			
Yes	17 (18.48%)	14 (16.09%)			
Smoking history, n (%)			0.496	1.762 (0.564–6.039)	0.338
No	5 (5.43%)	8 (9.20%)			
Yes	87 (94.57%)	79 (90.80%)			
Smoking cessation duration	2 [0; 37.5]	10 [0; 165]	0.066	1 (0.999–1)	0.072
Alcohol history, n (%)			0.698	1.451 (0.484–4.583)	0.507
No	6 (6.52%)	8 (9.20%)			
Yes	86 (93.48%)	79 (90.80%)			
Comorbidities, n (%)			0.302	0.951 (0.697–1.295)	0.747
No	57 (61.96%)	56 (64.37%)			
Hypertension	20 (21.74%)	16 (18.39%)			
Diabetes	10 (10.87%)	5 (5.75%)			
Others	5 (5.43%)	10 (11.49%)			
Family history, n (%)			0.017	10.48 (1.945–194.6)	0.027
No	82 (89.13%)	86 (98.85%)			
Yes	10 (10.87%)	1 (1.15%)			
Tumor Site, n (%)			0.853	0.938 (0.767–1.145)	0.528
Left upper lobe	24 (26.09%)	19 (21.84%)			
Left lower lobe	15 (16.30%)	16 (18.39%)			
Right upper lobe	28 (30.43%)	26 (29.89%)			
Right middle lobe	4 (4.35%)	2 (2.30%)			
Right lower lobe	21 (22.83%)	24 (27.59%)			
Tumor type, n (%)			0.999	0.932 (0.447–1.945)	0.851
Central	74 (80.43%)	69 (79.31%)			
Peripheral	18 (19.57%)	18 (20.69%)			
cT, n (%)			0.667	1.216 (0.869–1.711)	0.256
1	5 (5.43%)	5 (5.75%)			
2	32 (34.78%)	37 (42.53%)			
3	31 (33.70%)	28 (32.18%)			
4	24 (26.09%)	17 (19.54%)			
cN, n (%)			0.354	0.921 (0.566–1.491)	0.737
0	4 (4.35%)	6 (6.90%)			
1	36 (39.13%)	26 (29.89%)			
2	51 (55.43%)	55 (63.22%)			
3	1 (1.09%)	0 (0.00%)			
cTNM, n (%)			0.977	0.988 (0.658–1.483)	0.954
IIB	20 (21.74%)	18 (20.69%)			
IIIA	42 (45.65%)	41 (47.13%)			
IIIB	30 (32.61%)	28 (32.18%)			
Treatment cycle, n (%)			0.225	1.479 (0.95–2.34)	0.087
2	50 (54.35%)	57 (65.52%)			
3	30 (32.61%)	24 (27.59%)			
4	12 (13.04%)	6 (6.90%)			
Immunization drugs, n (%)			0.283	1.324 (0.896–1.977)	0.163
Keytruda	11 (11.96%)	18 (20.69%)			
Nivolumab	11 (11.96%)	9 (10.34%)			
Others	70 (76.09%)	60 (68.97%)			
Chemotherapeutic drugs, n (%)			0.158	0.502 (0.209–1.15)	0.11
Paclitaxel	82 (89.13%)	70 (80.46%)			
Others	10 (10.87%)	17 (19.54%)			
Platinum drugs, n (%)			0.768	0.917 (0.426–1.957)	0.823
Cisplatin	17 (18.48%)	14 (16.09%)			
Carboplatin	74 (80.43%)	73 (83.91%)			
Others	1 (1.09%)	0 (0.00%)			
Side effects stratification, n (%)			0.842	0.894 (0.481–1.656)	0.722
No	33 (35.87%)	29 (33.33%)			
Yes	59 (64.13%)	58 (66.67%)			
Side effects, n (%)			0.255	0.933 (0.704–1.234)	0.626
NO	33 (35.87%)	29 (33.33%)			
Myelosuppression	28 (30.43%)	21 (24.14%)			
Hepatic and renal function abnormalities	17 (18.48%)	27 (31.03%)			
Immune-related side effects	14 (15.22%)	10 (11.49%)			
Grading of side effects, n (%)			0.560	1.013 (0.772–1.332)	0.924
0	33 (35.87%)	29 (33.33%)			
1	26 (28.26%)	32 (36.78%)			
2	24 (26.09%)	18 (20.69%)			
3	6 (6.52%)	3 (3.45%)			
4	3 (3.26%)	5 (5.75%)			
Time interval	33.5 [29; 43.25]	34 [28; 40.5]	0.661	1.007 (0.989–1.027)	0.458
Time interval stratification, n (%)			0.624	1.263 (0.635–2.539)	0.507
≤42	68 (73.91%)	68 (78.16%)			
>42	24 (26.09%)	19 (21.84%)			
Pathology, n (%)			0.036	0.413 (0.194–0.81)	0.014
Squamous carcinoma	82 (89.13%)	65 (74.71%)			
Adenocarcinoma	9 (9.78%)	18 (20.69%)			
Others	1 (1.09%)	4 (4.60%)			

### 3.2 Univariate and multivariate analysis

Univariate analysis identified multiple factors associated with pCR, including pathological type, chemotherapy drug type, immunotherapy drug type, treatment cycle count, family history of cancer, smoking cessation duration, smoking index, and age (Table 1).

Multivariate logistic regression analysis revealed five independent predictors of pCR (p < 0.05) (Table 2):

1. Pathology: Non-squamous histology was associated with lower odds of pCR (OR 0.344 for non-squamous vs. squamous).
2. Family history of cancer: A positive family history significantly increased the likelihood of achieving pCR.
3. Smoking cessation duration: Shorter smoking cessation durations were associated with higher pCR rates.
4. Age: Older patients demonstrated slightly higher pCR rates.
5. Treatment cycle count: A higher number of treatment cycles correlated with increased pCR rates. Sensitivity analyses excluding family history (due to wide CI) or extreme smoking cessation durations yielded similar AUC (0.702), confirming model stability.

**TABLE 2:** Multivariate analysis for variables associated with pCR.

Variable	Multivariate analysis	
	OR (95% CI)	p-value
Age	1.053 (1.005–1.106)	0.032
Smoking cessation duration	0.999 (0.999–0.999)	0.033
Family history	10.76 (1.903–203.3)	0.027
Treatment cycle	1.621 (1.007–2.661)	0.049
Pathology	0.344 (0.151–0.707)	0.006

### 3.3 Receiver operating characteristic curve analysis, calibration analysis, decision curve analysis

**FIGURE 2:**
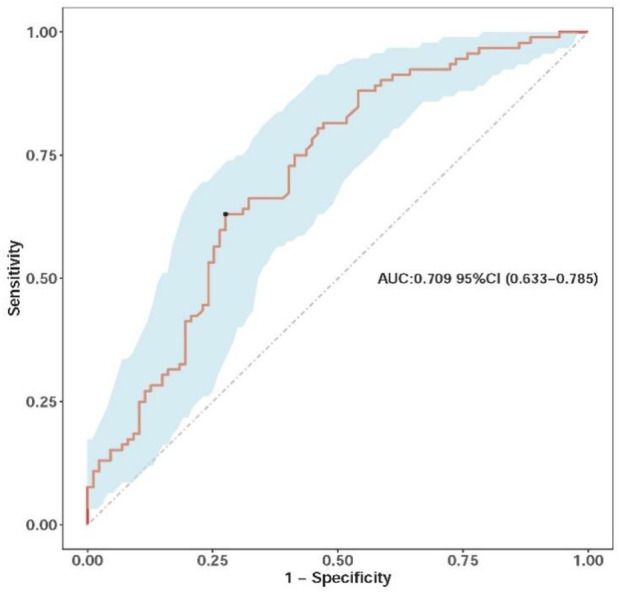
Receiver operating characteristic (ROC) curve of the nomograms, illustrating the model’s ability to discriminate between pCR and non-pCR cases, with the AUC value indicating modest discrimination.

The multivariate model demonstrated modest predictive performance, with an AUC of 0.709 (95% CI: 0.633–0.785) (bias-corrected AUC 0.692 via bootstrap, indicating minimal overfitting) (Figure 2).

**FIGURE 3:**
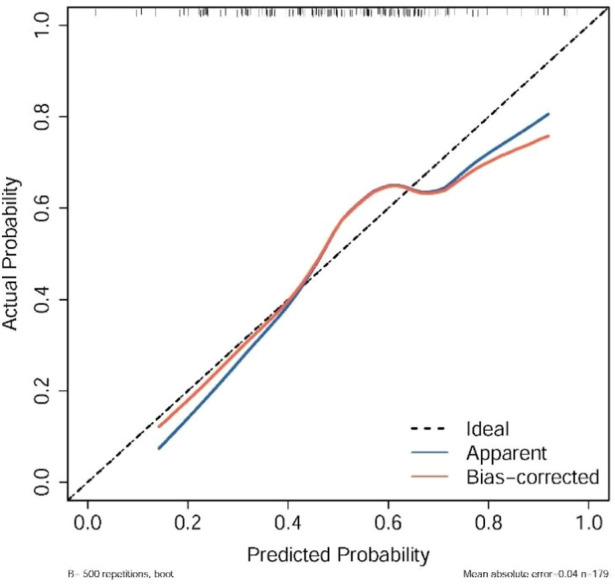
Calibration plots of the nomograms, showing agreement between predicted and observed pCR probabilities across bootstrap resamples, where the dashed line represents ideal calibration and points indicate observed vs. predicted bins.

The calibration curve indicated strong agreement between predicted probabilities and observed outcomes, particularly in most probability ranges, although minor deviations were noted between probabilities of 0.5 and 0.8. Internal validation using 500 bootstrap resamplings confirmed the model’s robustness (Figure 3).

**FIGURE 4:**
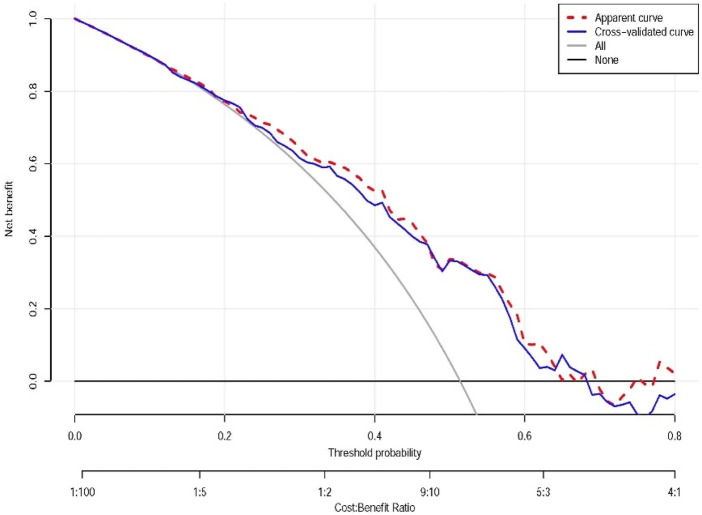
Decision curve analysis of the nomograms. Apparent curve: represented by the red dashed line, reflects the net benefit of the model on the training data. Cross-validated curve: represented by the blue dashed line, the net benefit curve obtained by the cross-validation method, providing a more reliable estimate of the model’s performance on new data. All: represented by the gray line, the net benefit assuming that all individuals take some action (e.g., all receive treatment or testing). All or nothing (None): represented by the horizontal line, the net benefit assuming that all individuals take no action. This plot demonstrates net clinical benefit across probability thresholds, with our model showing value at lower thresholds.

The decision curve analysis indicates that within a certain range of threshold probabilities, the model’s predictions can bring about net benefits, especially in the low threshold probability area. However, in the high threshold probability area, the model’s predictions may not be as good as taking no action at all (e.g., limited benefit above 0.6 threshold, suggesting primary utility for low-risk clinical decisions where avoiding overtreatment is prioritized). Through this analysis, we can determine the optimal threshold probability for the model in practical applications to maximize net benefits (Figure 4).

### 3.4 Nomogram

**FIGURE 5:**
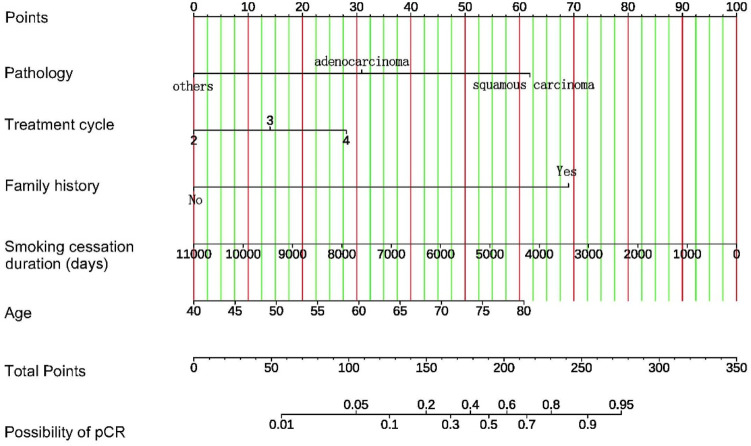
Nomogram for prediction of pCR after neoadjuvant chemotherapy combined with immunotherapy in patients with NSCLC. For each patient, five variables are assigned points on a nomogram, represented by five lines moving upward. The sum of these points is then located on the “Total Points” axis. A line is drawn downward from this point to predict the probability of achieving pCR, allowing individualized risk assessment.

**FIGURE 6:**
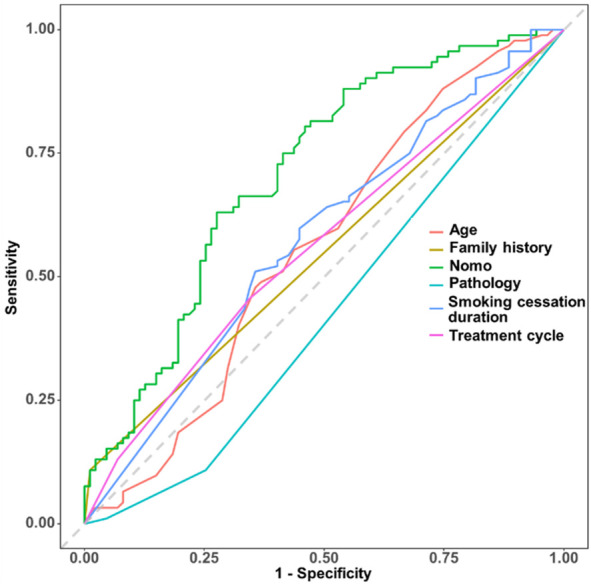
Rationality analysis based on model probability, comparing the model’s predictive performance (e.g., via AUC or other metrics) against individual variables to illustrate the nomogram’s superiority over single-factor predictions.

Based on the coefficients of multivariate logistic regression, a nomogram was established to predict pCR. The nomogram shows that if a patient scores higher total points, combining five variables, the predicted probability of pCR can be as high as 90% (Figure 5). The rationality analysis based on model probability (Figure 6) also shows that the model’s predictive ability is better than that of any single-factor variable.

## 4 Discussion

Identifying which non-small cell lung cancer (NSCLC) patients will benefit from neoadjuvant chemoimmunotherapy is crucial for optimizing treatment outcomes and avoiding unnecessary side effects (Topalian et al., 2020). This is particularly important given variability in response rates, driven by factors like histology and smoking history, as our study confirms (Qu et al., 2019). For instance, our finding that squamous carcinoma predicts higher pCR aligns with higher ICI sensitivity in this subtype due to greater TMB (Gandara et al., 2015).

One promising approach is the use of blood biomarkers to predict complete pathological response in NSCLC patients undergoing neoadjuvant chemoimmunotherapy. In the NADIM clinical trial, researchers identified specific immune parameters in peripheral blood that were associated with complete pathological response, suggesting that these biomarkers could be used to stratify patients before treatment (Laza-Briviesca et al., 2021). Our nomogram, while not incorporating blood biomarkers, could be integrated with such tools for enhanced prediction, as family history in our model may proxy genetic susceptibility (de Alencar et al., 2020), though the wide CI for family history (1.903–203.3) indicates potential instability due to low event rates (n = 11 positive cases); sensitivity analysis excluding this variable yielded a similar AUC (0.702), confirming model stability.

Researchers have also turned to advanced imaging techniques to predict treatment success. A deep learning model built on computed tomography (CT) imaging data has shown remarkable accuracy in predicting pathologic complete response to neoadjuvant chemoimmunotherapy in NSCLC. This innovation highlights how imaging data could become a key tool in identifying suitable candidates for this aggressive treatment strategy (Qu et al., 2024). Similarly, our clinical-variable nomogram complements imaging by providing a low-cost, immediate alternative where advanced CT analysis is unavailable.

While prior studies have explored delta-radiomics for superior prediction (Han et al., 2024), our focus on clinical variables directly supports current findings without relying on such advanced features.

In a single-center study, scientists developed a predictive score for major pathological response using pre-treatment parameters such as prothrombin time, neutrophil percentage, and PD-L1 expression. This model offers a practical framework for personalized decision-making in operable NSCLC treated with neoadjuvant chemoimmunotherapy (Hu et al., 2024). Our model, unlike (Hu et al., 2024), excludes PD-L1 but includes modifiable factors like smoking cessation. Our inclusion of smoking cessation duration extends this by highlighting modifiable factors; shorter durations correlated with higher pCR, though this association is speculative and may reflect residual immune activation or confounding factors; it conflicts with known OS benefits of long-term cessation (Bokemeyer et al., 2023) and requires mechanistic and prospective studies for confirmation. This counterintuitive finding may arise from residual inflammation enhancing immunotherapy response or bias in our smoking-dominant cohort.

The tumor immune microenvironment also plays a significant role in predicting the pathologic response of neoadjuvant chemoimmunotherapy in NSCLC. A study found that specific patterns of tumor-infiltrating lymphocytes were associated with favorable pathological responses, underscoring the value of immune profiling in treatment planning (Han et al., 2023). While our study did not assess microenvironment, the higher pCR with more cycles suggests prolonged exposure enhances immune activation, linking to these findings (Cascone et al., 2021).

Insights from a meta-regression analysis of randomized clinical trials reveal that the benefits of neoadjuvant chemotherapy in NSCLC are most pronounced in stage 3 disease when protocols include three chemotherapeutic agents. This finding reinforces the importance of aligning treatment strategies with disease stage and chemotherapy protocols (Bozcuk et al., 2012). Our results support this, with more cycles (up to 4) improving pCR odds (OR = 1.621), but we caution on toxicity risks in older patients, another predictor in our model. The positive association with older age may reflect selection bias (fitter elderly patients selected for treatment) or age-related tumor biology differences, contrasting with expectations of stronger immunity in younger individuals (Liao et al., 2024); this requires further validation.

These studies collectively emphasize the importance of predictive biomarkers and advanced imaging techniques in identifying NSCLC patients who are likely to benefit from neoadjuvant chemoimmunotherapy, thereby enhancing treatment efficacy and minimizing unnecessary exposure to potential side effects. Our nomogram bridges this by using routine data, achieving moderate discrimination (AUC = 0.709) and good calibration, with DCA showing net benefit at low thresholds, though the modest AUC limits its standalone use and highlights the need for integration with other tools.

Using real-world clinical data, this study successfully developed a nomogram model to predict pathological complete response (pCR) in NSCLC patients receiving neoadjuvant chemotherapy combined with immunotherapy. By retrospectively collecting data from patients treated between 2019 and 2022, the model was constructed through an in-depth analysis of clinical parameters, including age, gender, smoking history, comorbidities, neoadjuvant treatment regimens, and adverse reactions. Multivariate analysis identified five significant predictors of pCR: pathological type, family history of cancer, smoking cessation duration, age, and the number of neoadjuvant treatment cycles. These findings provide new insights into the factors influencing pathological responses in NSCLC patients undergoing combination therapy.

### 4.1 Key predictive factors

Pathological type emerged as a pivotal predictor of pCR. NSCLC subtypes, including squamous cell carcinoma (SCC) and adenocarcinoma, exhibit distinct molecular features, biological behaviors, and treatment sensitivities. SCC demonstrates higher sensitivity to immunotherapy, likely due to its unique molecular characteristics (Chi et al., 2021; Drilon et al., 2012). Studies indicate that ICIs yield significant therapeutic benefits in SCC patients, though their response to traditional chemotherapy may be less robust (Kuribayashi et al., 2016). Recent advancements in immunotherapy and targeted therapies offer new hope for improving SCC outcomes (Santos and Rodriguez, 2022). Conversely, adenocarcinoma tends to respond more favorably to platinum-based chemotherapy, potentially due to molecular characteristics such as epidermal growth factor receptor (EGFR) mutations (Reungwetwattana et al., 2011; Wang et al., 2021). Targeted therapies for EGFR and ALK mutations have further enhanced the prognosis of adenocarcinoma patients (Naylor et al., 2016). Incorporating pathological type into the prediction model allows for more accurate assessment of a patient’s likelihood of achieving pCR.

The role of family history as a predictive factor underscores the influence of genetic predisposition on NSCLC development and progression. A family history of NSCLC suggests genetic susceptibility, with studies identifying rare variants associated with increased disease risk. These variants, often related to tumor suppressor gene function or DNA repair pathways, may play a critical role, particularly in non-smokers or patients with non-squamous subtypes (Dudbridge et al., 2018; Miyabe et al., 2023). Family history may proxy genetic TMB, enhancing ICI response; this underexplored association warrants further genetic studies to confirm if it acts as a surrogate for factors like TMB or inherited variants.

Smoking cessation duration also proved to be a significant factor. Long-term smoking is a well-established risk factor for NSCLC, but quitting smoking can improve lung function, reduce inflammation, and enhance treatment outcomes (Perret and Walters, 2020). The longer a patient has abstained from smoking, the greater the improvement in lung function and overall survival, highlighting the positive impact of lifestyle changes on therapeutic efficacy (Bokemeyer et al., 2023; de la Rosa-Carrillo et al., 2024). However, our research findings show that the longer the smoking cessation duration, the lower the probability of pCR, which also suggests that pCR does not necessarily translate into long-term OS. This phenomenon deserves further in-depth study. This counterintuitive association may reflect residual immune activation from recent smoking or confounding by unmeasured factors such as pack-years; it conflicts with known OS benefits of long-term cessation and requires mechanistic studies. Age was another significant predictor, likely reflecting physiological reserves, immune status, and treatment tolerance. Interestingly, older age was associated with higher pCR rates, which may reflect selection bias toward fitter elderly patients or differences in tumor biology; this contrasts with expectations of stronger immunity in younger patients and requires prospective validation (Liao et al., 2024).

The number of neoadjuvant treatment cycles also significantly influenced pCR. Extended treatment cycles provide more thorough therapy, increasing the likelihood of complete tumor remission. However, prolonged treatment may also heighten toxicity risks, necessitating a careful balance between efficacy and patient tolerance when designing individualized treatment plans (Wang et al., 2024).

### 4.2 Clinical implications

The nomogram developed in this study integrates multiple clinical parameters to provide a comprehensive and accurate tool for predicting pCR in NSCLC patients. Compared to traditional methods based on clinical staging or single biomarkers, the nomogram offers superior predictive performance. In practice, clinicians can use this tool to assess a patient’s likelihood of achieving pCR, enabling more precise and personalized treatment planning. High predicted pCR (>60%) may support proceeding to resection; low scores (<40%) could prompt biomarker testing or alternative regimens. From a thoracic surgical perspective, this aids in operative planning, potentially avoiding futile resections in low-pCR probability cases.

### 4.3 Limitations and future directions

This study has several limitations. First, as a single-center retrospective analysis with a relatively small sample size, the generalizability of the findings is limited, particularly given the male-dominant cohort (95.5%), which may bias results toward smoking-related factors common in male populations in our region. The cohort is male-dominant (95.5%), which may reflect the higher incidence of NSCLC in male smokers in our region but limits generalizability to female patients, potentially biasing results toward smoking-related factors. The high pCR rate (51.4%) may not reflect broader populations, and the lack of external validation raises overfitting concerns, though mitigated by bootstrap (bias-corrected AUC 0.692). Additionally, the wide confidence intervals (e.g., for family history) suggest instability due to low event rates, and sensitivity analyses excluding extreme smoking cessation durations confirmed model stability but highlight the need for larger cohorts. The absence of biomarkers like PD-L1, TMB, or EGFR status-prevalent in Asian populations (Shi et al., 2015)-limits comprehensiveness. The lack of external validation is a major limitation, as internal bootstrap alone may not fully address overfitting (Harrell’s C-index 0.709, bias-corrected 0.692). External validation using data from other institutions or prospective studies is required. Second, the study primarily focused on clinical parameters and excluded multi-omics data such as radiomics, genomics, and molecular pathology (e.g., PD-L1, EGFR status, common in Asian NSCLC (Shi et al., 2015)), which could enhance the model’s predictive power. Finally, although internal validation using the bootstrap method confirmed the model’s robustness, external validation remains necessary to establish its reliability. Future multi-center studies should externally validate the model and integrate omics data (e.g., PD-L1, TMB) for improved performance.

## 5 Conclusions

This nomogram, derived from routine clinical data, predicts pCR after neoadjuvant chemoimmunotherapy in NSCLC, offering a tool for thoracic surgeons to optimize treatment and surgical planning. However, external validation is required before clinical application, particularly in diverse populations to address generalizability.

## Data Availability

The original contributions presented in the study are included in the article/supplementary material, further inquiries can be directed to the corresponding authors.
